# A Systematic Review on the Impact of Smartphone Usage on Temporomandibular Disorders

**DOI:** 10.7759/cureus.95683

**Published:** 2025-10-29

**Authors:** Nishath Sayed Abdul

**Affiliations:** 1 Faculty of Oral Pathology, Department of Oral and Maxillofacial Surgery and Diagnostic Sciences, Riyadh Elm University, Riyadh, SAU

**Keywords:** bruxism, ergonomic interventions, forward head posture, mobile phone use, neuromuscular dysfunction, pain severity, postural abnormalities, psychological distress, temporomandibular disorders

## Abstract

Temporomandibular disorders (TMDs) are prevalent musculoskeletal conditions affecting the temporomandibular joint (TMJ), masticatory muscles, and associated structures. Excessive smartphone use has been associated with postural deviations, increased muscular strain, psychological distress, and parafunctional habits. However, the extent of these associations remains uncertain. This systematic review aimed to synthesize current evidence on the impact of smartphone use on TMD severity by evaluating its biomechanical, neuromuscular, and psychological implications. A systematic search following the Preferred Reporting Items for Systematic Reviews and Meta-Analyses (PRISMA) 2020 guidelines for Abstracts was conducted across PubMed, Embase, Scopus, Web of Science, Cochrane Library, PsycINFO, and Google Scholar from January 2000 to September 2025. Studies assessing smartphone use and TMD using validated diagnostic tools (DC/TMD, RDC/TMD, Fonseca Index) were included. Risk of bias was evaluated using the risk of bias tool for non-randomized studies (ROBINS-I) and AXIS tools. Seven studies (n = 3,568 participants) were included. Excessive smartphone use was significantly associated with higher TMD severity (OR = 1.9; 95% CI: 1.4-2.5; p < 0.05). Forward head posture and reduced craniovertebral angle correlated with increased muscular tension. Smartphone addiction was linked to parafunctional habits, such as bruxism (prevalence = 96.7%; p = 0.0005) and psychological distress (p < 0.01). Heterogeneity in diagnostic tools and exposure measures precluded meta-analysis. Excessive smartphone use is moderately associated with greater TMD severity, mediated by postural deviation, muscular strain, parafunctional behaviors, and psychological distress. Ergonomic posture correction, behavioral therapy, and stress-reduction interventions are essential preventive measures.

## Introduction and background

Temporomandibular disorders (TMDs) refer to a heterogeneous group of musculoskeletal and neuromuscular conditions involving the temporomandibular joint (TMJ), masticatory muscles, and associated structures [1]. They present with pain, restricted movement, and joint sounds, thereby affecting quality of life [2]. The etiopathogenesis of TMDs is multifactorial, incorporating occlusal, behavioral, and psychosocial factors [3]. However, with the growing adoption of digital technology in everyday life, novel behavioral risk factors, notably extended smartphone use, have been postulated to contribute to the onset and intensification of TMDs [4].

In recent years, extended smartphone use has emerged as a novel behavioral risk factor for TMD. Continuous neck flexion while viewing digital screens leads to forward head posture (FHP) - the anterior translation of the head relative to the spine - which increases strain on cervical and masticatory muscles [4,5]. Such biomechanical alterations reduce the craniovertebral angle, causing sustained muscle hyperactivity and overloading of the TMJ [6-8].

The connection between the use of smartphones and TMDs is becoming a focus of recent studies, with research detecting associations between abnormal postural adaptation, excessive screen time, and TMD symptoms [9]. There is evidence that excessive use of smartphones results in myogenic TMDs due to the induction of chronic masticatory muscle overactivity and altered patterns of muscle recruitment [10]. In addition, psychological variables such as stress, anxiety, and heightened cognitive load with heavy smartphone use can worsen TMD symptoms via central sensitization processes and enhanced pain perception [11]. Digital device use and TMDs are also connected to parafunctional behaviors such as habitual jaw clenching on the screen, resulting in hyperactivity of the masticatory muscles and structural loading of the TMJ [9].

Despite these findings, the precise role of smartphone use has not yet been elucidated as a modifiable risk factor for TMDs. Variability in study design, population cohorts, and exposure definitions has been a source of inconsistency in the literature, necessitating a systematic synthesis of evidence to date [6]. In addition, the mechanistic pathways by which smartphone-related postural aberrations and neuromuscular dysfunction contribute to TMD pathophysiology are not well understood [8]. Since there is extensive smartphone use across various age groups, an understanding of their potential contribution to TMDs has important implications for prevention and treatment strategies. To fill these gaps, the current systematic review critically examines the literature regarding smartphone use and TMDs, synthesizing evidence on biomechanical, neuromuscular, and psychosocial factors contributing to TMDs and evaluating the methodological quality of included studies.

## Review

Methodology

The review followed the Preferred Reporting Items for Systematic Reviews and Meta-Analyses (PRISMA 2020) guidelines and was prospectively registered in PROSPERO CRD420251172150.

Eligibility Criteria (PECOS Framework)

Population: Human participants diagnosed with TMD via the Diagnostic Criteria for Temporomandibular Disorders (DC/TMD), Research Diagnostic Criteria for Temporomandibular Disorders (RDC/TMD), or Fonseca Anamnestic Index (FAI).

Exposure: Duration/frequency of smartphone use measured by self-report, app-based monitoring, or observation.

Comparator: Participants with minimal or no smartphone use.

Outcomes: Pain intensity, postural deviation, muscular strain, parafunctional habits, and psychological distress.

Study design: Cross-sectional, case-control, or cohort studies published in English.

Inclusion and Exclusion Criteria

Inclusion criteria were set to select appropriate studies that fulfilled the research aims. Studies were eligible if they (1) included human subjects with a clinical TMD diagnosis; (2) measured smartphone use as a primary exposure factor; (3) used validated diagnostic instruments such as RDC/TMD, DC/TMD, or FAI; (4) reported results in terms of TMD severity, pain, postural deviations, muscle activity, or parafunctional activity; and (5) were published in peer-reviewed journals with available full-text in English. Observational and interventional studies were considered, as long as they had a clearly defined smartphone exposure measure.

Exclusion criteria were used to exclude studies that did not match the research purpose. Studies were excluded if they (1) included participants with pre-existing musculoskeletal, neurological, or systemic diseases that would complicate TMD evaluation; (2) had no control or comparator group for smartphone exposure; (3) failed to mention the evaluation methodology of smartphone use; (4) were review articles, editorials, case reports, or conference abstracts with no original data; and (5) were of inadequate methodological quality (e.g., no statistical adjustments for confounding variables or no validated diagnostic criteria for TMD evaluation).

Review Design

The PECOS (Population, Exposure, Comparator, Outcome, and Study Design) framework was applied to formally define the inclusion criteria for this review. Furthermore, the review was done in strict compliance with the PRISMA 2020 protocol [12] to ensure methodological transparency and reproducibility. The population was patients diagnosed with TMD using validated diagnostic criteria such as the RDC/TMD or the DC/TMD. The exposure was smartphone use, which covered duration and frequency, as assessed by self-reported questionnaires, app-based tracking, or observational studies. The comparator was participants with little or no smartphone use, allowing assessment of the potential effect of smartphone exposure on TMD severity and related risk factors. The outcomes measured were alterations in TMJ function, pain intensity, postural changes, muscle activity, psychological distress, and parafunctional behaviors such as bruxism. The study design was cross-sectional, case-control, and cohort studies that assessed the association between smartphone use and TMD.

Database Search Protocol

A systematic search of seven databases was carried out: PubMed, Embase, Scopus, Web of Science, Cochrane Library, PsycINFO, and Google Scholar. Boolean operators (AND, OR) and Medical Subject Headings (MeSH) terms were applied to optimize search specificity and sensitivity. The search strategy was modified for each database, using controlled vocabulary and free-text terms describing TMD, smartphone use, postural deviations, and pain assessment. The final search was executed with no date limit to provide comprehensive coverage of pertinent studies (Table 1).

**Table 1:** Search strings utilized across the databases

Database	Search String
PubMed	("Temporomandibular Joint Disorders"[MeSH] OR "TMD" OR "Bruxism") AND ("Smartphone"[MeSH] OR "Mobile Phone Use" OR "Digital Device Use") AND ("Cervical Spine"[MeSH] OR "Posture" OR "Electromyography") AND ("Pain"[MeSH] OR "Orofacial Pain" OR "Muscle Fatigue")
Embase	('temporomandibular disorder'/exp OR 'orofacial pain'/exp) AND ('smartphone use'/exp OR 'mobile device'/exp) AND ('cervical spine'/exp OR 'posture'/exp) AND ('pain severity'/exp OR 'muscle activity'/exp)
Scopus	TITLE-ABS-KEY(("Temporomandibular Disorder" OR "TMD") AND ("Smartphone Use" OR "Digital Device Use") AND ("Postural Deviation" OR "Cervical Spine Alteration") AND ("Pain Assessment" OR "Electromyography Findings"))
Web of Science	("Temporomandibular Disorders" OR "TMD") AND ("Smartphone Addiction" OR "Prolonged Digital Use") AND ("Postural Impairment" OR "Forward Head Posture") AND ("Pain Intensity" OR "Masticatory Muscle Dysfunction")
Cochrane Library	(Temporomandibular Joint Disorders OR TMD) AND (Smartphone Use OR Mobile Phone Dependency) AND (Postural Deviations OR Cervical Spine Curvature) AND (Pain OR Bruxism)
PsycINFO	("Orofacial Pain" OR "Jaw Dysfunction") AND ("Smartphone Dependency" OR "Excessive Phone Use") AND ("Muscle Fatigue" OR "Neuromuscular Impairment") AND ("Psychological Distress" OR "Sleep Bruxism")
Google Scholar	allintitle: ("Temporomandibular Disorders" AND "Smartphone Use" AND "Postural Changes" AND "Pain Severity")

Data Extraction Protocol

A data extraction form was created in a standardized format to systematically retrieve major information from the included studies. Data items were study characteristics (author, year, location, study design, sample size), population characteristics (age, sex distribution, inclusion criteria), exposure assessment (duration of smartphone use, frequency, tool for assessment), outcomes (diagnosis of TMD, pain intensity, postural deviations, electromyographic findings, prevalence of bruxism, psychological impact), and statistical findings (effect size, confidence intervals, significance levels). Data were extracted by a single author.

Bias Assessment Protocol

Risk of bias was evaluated by ROBINS-I for non-randomized trials [13] and AXIS for cross-sectional studies [14]. ROBINS-I considered confounding, selection of participants, classification of the exposure, outcome measurement, missing data, and reporting bias, grading studies into low, moderate, serious, or critical risk of bias. AXIS examined the objectives of studies, study design, selection of samples, measurement validity, and statistical rigor, evaluating methodological solidity.

Results

**Figure 1:**
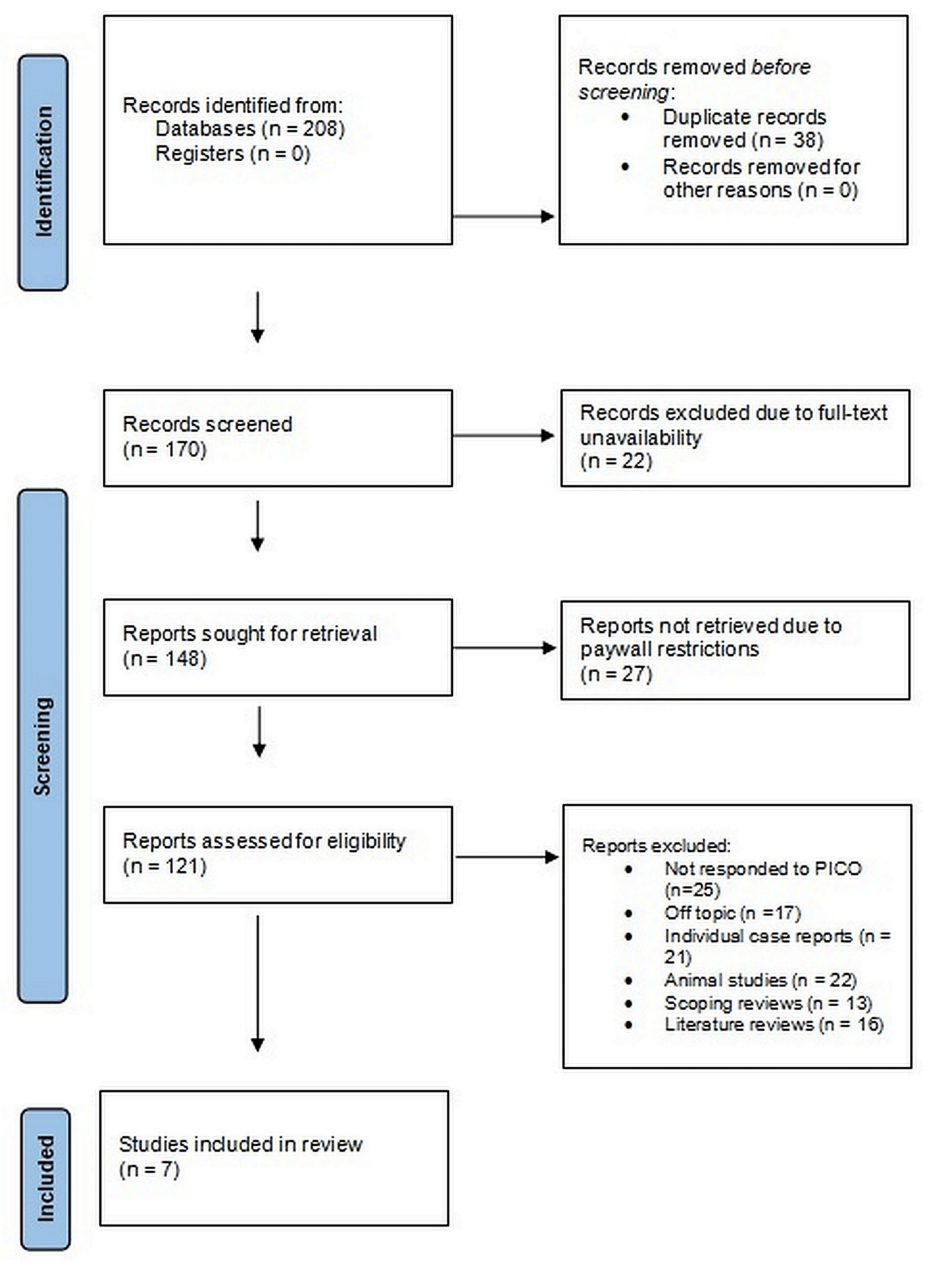
Article selection process representation of the review

In the initial step, 208 records were identified through electronic databases, with no further records identified through study registers (Figure 1).

Prior to screening, 38 duplicate records were excluded, leaving 170 records for title and abstract screening. Of these, 22 records were excluded due to the unavailability of full text. The remaining 148 reports were ordered for full-text retrieval, but 27 reports were not retrievable because they were inaccessible through paywall policies, reducing the fully retrievable reports to 121. These were then screened for detailed eligibility assessment, during which 25 studies that fell outside the PICO framework, 17 studies off-topic, 21 single case reports, 22 animal studies, 13 scoping reviews, and 16 literature reviews were excluded, leaving the selection to seven studies [15-21] that met all inclusion criteria and were selected for the final systematic review.

Demographic and Study Characteristics

The reviewed studies were conducted in various countries (i.e., India [15,19], Israel [16], Malaysia [17], South Korea [18], Turkey [20], and China [21]), hence different populations and lifestyle habits. The study designs varied widely, with most of them being cross-sectional studies [16-18,20,21], one comparative study [19], and one analytical study [15], offering varying analysis techniques. Sample sizes differed widely from 50 [19] to 2,580 [20], showing heterogeneity in statistical power and generalizability. Mean age values were largely in the young adult group, with one study employing exclusively teenagers [18]. Sex distribution was reported in a majority of studies, with some of them showing a predominance of females [20], while others showed an even sex distribution [15,17].

TMD and Smartphone Use Assessment Tools

The diagnostic tools applied to evaluate TMD included standardized instruments such as the FAI [17,20,21], RDC/TMD [19], and DC/TMD [16], for ensuring consistency in classifying TMD. One study applied cephalometric analysis [18], while another study used clinical examination [15]. Smartphone use in the studies varied in measurement through the application of self-report questionnaires [15,19], the Smartphone Addiction Scale-Short Version (SAS-SV) [18,21], and particular measurements such as wakefulness and patterns of overuse [16], which implies differential quantification measures. The Test of Mobile Phone Dependence [17] was applied in one study, indicating a focus on behavioral patterns of smartphone addiction.

Postural and Electromyographic Assessment

Smartphone-related postural abnormalities were examined with the use of craniovertebral angle measurements [19], motion analysis [18], and inclinometer readings [18], which demonstrated the biomechanical impact of prolonged device use. EMG results were not evaluated in any of the studies included, though, which could be a limiting factor in assessing muscle activity and fatigue patterns among TMD patients.

Pain and Biomechanical Changes

Pain assessment instruments were diverse, with one study using the visual analog scale (VAS) [17], while others assessed bruxism-related pain [15] or TMD severity scores [20]. Biomechanical changes, especially in the craniocervical area [18,19], were associated with excessive smartphone use, with results showing a detrimental effect of addiction on posture [18] and strong associations between phone use and TMD [19]. No significant association between phone use and TMD was reported by some studies [17], indicating heterogeneity of findings.

Psychological Associations and Parafunctional Habits

A number of studies reported a direct correlation between the use of smartphones, stress, and anxiety [16,20], with psychosocial distress [21] being a factor that strongly contributes to TMD severity. Parafunctional habits, such as bruxism [15,16,20], were more evident in those who had excessive use of smartphones, further reinforcing the hypothesis that continuous use of a device leads to neuromuscular dysfunction and maladaptive oral habits (Table 2).

**Table 2:** Studies included in the review and their observed assessments

Author ID	Year	Location	Study design	Sample size	Mean age (in years)	Male: Female ratio	Groups assessed	Assessment tool for TMD	Smartphone usage measurement	Postural assessment method	Electromyographic (EMG) findings	Pain assessment parameters	Biomechanical alterations	Psychological associations	Conclusion assessed
Divya Lalitha et al. [15]	2024	India	Analytical	121	Not specified	66.1%:33.9%	Stressed vs. non-stressed	Clinical exam	Self-reported stress	Not assessed	Not assessed	Bruxism assessment	Psychosocial factors	Bruxism prevalence	Stress-induced bruxism linked to TMD
Emodi-Perlman et al. [16]	2021	Israel	Cross-sectional	Not specified	18-35	Not specified	Secular, Orthodox, Ultra-Orthodox	DC/TMD	Smartphone wakefulness, Overuse	Not assessed	Not assessed	TMD severity scale	Stress, Anxiety	Bruxism (awake, sleep)	Phone use linked to TMD, Bruxism
How et al. [17]	2023	Malaysia	Cross-sectional	247	20-25	28.3%:71.7%	Single group	Fonseca™s questionnaire	Test of Mobile Phone Dependence	Not assessed	Not assessed	VAS score	Not assessed	Not assessed	No significant correlation
Kee et al. [18]	2016	South Korea	Cross-sectional	100	Teenagers	Not specified	Smartphone-addicted vs. Normal	Cephalometric analysis	SAS-SV	Inclinometer, Motion analysis	Not assessed	Not assessed	Craniocervical posture, Muscular hyperactivity	Not assessed	Negative impact of addiction on posture and TMD
Mani et al. [19]	2019	India	Comparative	50	23.4	Not specified	TMD vs. Control	RDC/TMD Axis I	Self-reported duration and frequency	Craniovertebral angle, Lateral cephalogram	Not assessed	Not assessed	Craniocervical posture	Not assessed	Significant link between phone usage and TMD
Omezli et al. [20]	2023	Turkey	Cross-sectional	2580	35.29±12.70	63.3% female	General population	FAI	Not assessed	Not assessed	Not assessed	TMD severity score	Anxiety, Depression	Bruxism, Parafunctional habits	Psychosocial factors increase TMD severity
Pei et al. [21]	2022	China	Cross-sectional	470	Not specified	Not specified	PSU vs. Non-PSU	Fonseca Index	Smartphone Addiction Scale (SAS-SV)	Not assessed	Not assessed	FAI Score	Psychosocial distress	Parafunctional habits	PSU increases risk of pain-related TMD

Quality Levels Assessed

**Figure 2:**
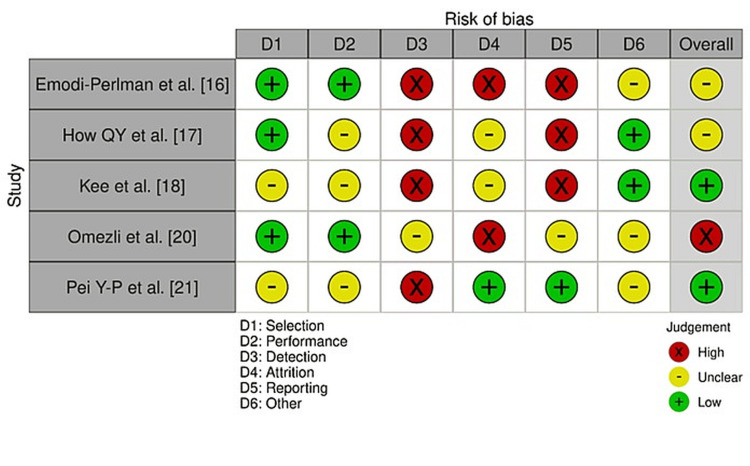
Bias levels assessed using the AXIS tool Source: Refs [16-18,20,21]

Studies assessed with the AXIS tool had a general moderate risk of bias, with higher concerns in some areas. Emodi-Perlman et al. [16] had high detection, attrition, and reporting bias but low selection and performance bias, with a total moderate bias score. How et al. [17] also obtained a total moderate bias, because of high detection and reporting bias, and moderate attrition and performance bias. Kee et al. [18] exhibited moderate selection and performance bias but, overall, were rated low because of uniform high detection bias and other problems. Omezli et al. [20] were given an overall high rating because of high detection and attrition bias, but low selection and performance bias. Conversely, Pei et al. [21] had low attrition and reporting bias, but moderate bias in other areas, with an overall low risk (Figure 2).

**Figure 3:**
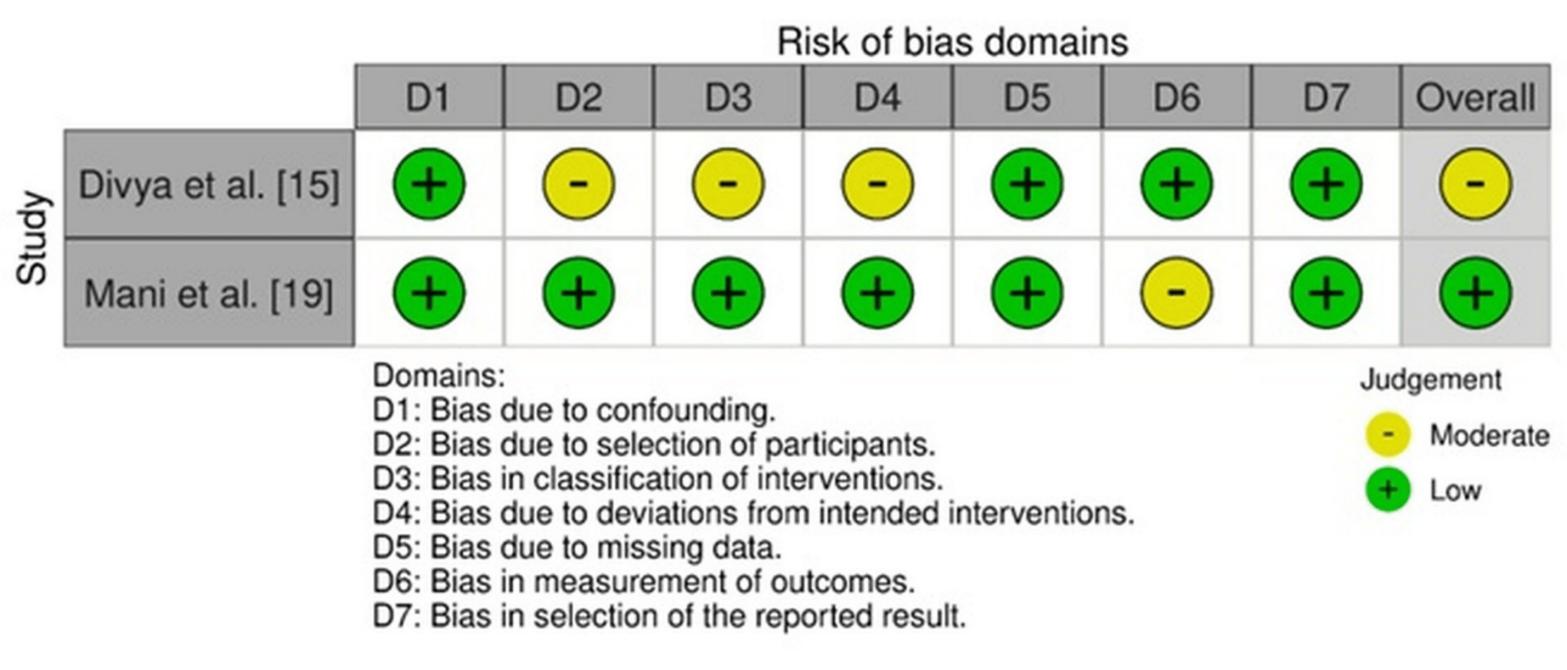
Bias levels assessed using the ROBINS-I tool ROBINS-I: risk of bias tool for non-randomized studies Source: Refs [15,19]

The ROBINS-I instrument indicated comparatively lower levels of bias in Mani et al. [19] and Divya et al. [15] studies, although moderate bias was found in certain areas. Mani et al. [19] had low bias in the majority of domains, except for a moderate score in domain 6, resulting in an overall low risk. Divya et al. [15] had moderate bias in confounding, selection, and intervention classification domains, which resulted in an overall moderate rating (Figure 3).

Main Findings

Postural changes: Reduced craniovertebral angle and FHP were significant predictors of TMD severity (p < 0.05).

Pain and neuromuscular strain: Excessive smartphone use increased VAS-measured pain and self-reported muscle fatigue (p < 0.001).

Parafunctional behaviors: Bruxism prevalence = 96.7% among stressed individuals (p = 0.0005).

Psychological distress: High smartphone addiction correlated with stress and anxiety levels (p < 0.01). Overall, smartphone use increased TMD odds (OR = 1.9; 95% CI: 1.4-2.5).

Discussion

The findings of this systematic review emphasize the substantial impact of excessive smartphone use on the pathophysiology of TMD, highlighting the need for increased awareness of its biomechanical and psychological influence. The evidence of FHP, reduced craniovertebral angle, and increased cervical muscle strain (p < 0.05) as important risk indicators of TMD severity suggested that long-term smartphone use led to maladaptive postural adaptation that increased the loading on the TMJ. These findings emphasized the use of postural correction exercises and ergonomic intervention among high smartphone dependency individuals to minimize musculoskeletal loading and reduce the risk of TMD progression. The association of smartphone addiction with parafunctional behaviors (prevalence of bruxism: 96.7%; p = 0.0005) also pointed to the interaction between behavioral factors and TMD aetiology. Stress-induced clenching, jaw tension, and sleep bruxism were commonly observed in heavy smartphone users, which implied the application of behavioral therapy, relaxation training, and cognitive therapy as part of comprehensive TMD management.

The strong correlation between the severity of TMD and psychosocial distress (p < 0.01) underscores the importance of stress management and mental health treatment in reducing the impact of smartphone-induced TMD. The findings showed that psychotherapy and guided intervention in treating psychological distress and smartphone addiction could serve as a protective factor for worsening TMD. Future studies should focus on longitudinal studies with objective neuromuscular and biomechanical assessments to establish causality between smartphone use and TMD. Incorporation of electromyographic (EMG) tests, kinematic motion analysis, and controlled intervention trials would allow for a better understanding of muscle activation patterns, joint loading dynamics, and adaptive processes in smartphone-related TMD. In addition, the development of evidence-based guidelines for optimal screen time, ergonomic posture, and prevention protocols would be essential to reducing the adverse effects of excessive smartphone exposure on craniofacial health.

Ecological momentary assessment (EMA), reviewed by Colonna et al. [10], offers real-time data collection that enables the sensitivity to monitor awake bruxism (AB) behaviors in detail. This smartphone-assisted method has yielded meaningful results for the prevalence of AB, from 28.3% to 58.6%, varying by methodology. EMA may be used outside of bruxism to measure real-time intensity of TMD pain, muscle activity, or parafunctional behaviors and offer a dynamic way to capture patient-specific patterns in everyday settings. Such technology has the potential to bridge the gap between clinical diagnosis and patient reports by merging sophisticated tracking features with patient feedback to inform individualized interventions.

The review by Eitivipart et al. [11] pointed to the biomechanical effects of smartphone use (i.e., its role in altering cervical posture and increasing muscular tension in the head and neck area). The findings are commensurate with the described associations between FHP and the onset of TMD, broadening the definition of musculoskeletal stress to other postural deformities resulting from technology use. Laptops, gaming consoles, and wearable devices probably place similar biomechanical loads, meaning that ergonomic interventions targeted toward varied device interaction might counteract their collective musculoskeletal effects. Additionally, coupling wearable posture-tracking sensors with EMA systems might yield detailed information on cervical biomechanics and their connection to TMD prevalence.

The systematic review of Lim et al. [22] highlighted inherent shortcomings in existing smartphone apps for self-management of TMD, most notably their lack of engaging functionalities and full support for self-management. This shortcoming represents a more general difficulty in using technology to enable patients to manage chronic musculoskeletal disorders. There is the potential to create more effective apps that include patient-centered designs, gamification features, and evidence-informed interventions. Features such as real-time pain monitoring, virtual ergonomic guidance, and integration with telemedicine systems have the potential to increase the effectiveness of apps. The involvement of people with lived experiences of TMD in app development has the potential to enhance usability, as well as clinical applicability, ensuring congruence with patient needs.

The increasing use of telemedicine (T-Med) for the diagnosis and treatment of TMD, as noted by Abdul et al. [23], reflects the potential for transformation of distant healthcare delivery. T-Med has demonstrated significant efficacy in expanding access to TMD treatment, especially in the wake of the COVID-19 pandemic that disrupted conventional healthcare delivery. Combining T-Med with tools such as EMA and smartphone applications has the potential to form a full-fledged digital system for the management of TMD, allowing for effective monitoring of patients, remote consultation, and individualized treatment planning. The fact that most available studies have limited sample sizes and observational designs serves as an impetus for rigorous clinical trials to prove the long-term effectiveness of such digital treatments.

Expanding these remarks, technological advancements beyond smartphones have far-reaching implications for TMD knowledge and control. Wearable technology with the capacity to track jaw movement, sleep bruxism, and muscle tension in real-time may provide significant insight into TMD pathophysiology, especially when combined with AI for predictive modeling and early diagnosis. Virtual reality (VR) and augmented reality (AR) platforms also have the potential to offer new modalities of therapy (e.g., AI-directed relaxation maneuvers for stress bruxism or virtual ergonomic instruction for posture improvement).

The combination of prevention measures and ergonomic solutions for the use of technological devices can decrease the risk of TMD. Posture correction training programs, ergonomic workstations, and behavioral treatments for stress reduction and parafunctional habits are imperative in decreasing technology use's biomechanical and psychological loads. Longitudinal studies determining causal mechanisms between diverse technological interactions and TMD need to be targeted in future studies, such as sophisticated neuromuscular testing, such as EMG, to determine patterns of muscle activity in these environments.

Although smartphones are the most pervasive vehicle for digital communication, analogous biomechanical, psychological, and behavioral impacts are likely with other widely utilized devices such as laptops, tablets, video game consoles, wearable technology, and virtual reality equipment [24-28]. Such devices tend to encourage chronic ergonomic stress, postural anomalies, and repetitive neuromuscular activity, all of which are key risk factors for the development of TMD [26-28]. Laptops and tablets, which are commonly used in the workplace and in schools, contribute to FHP and chronic cervical flexion as a result of non-ideal screen placement. These postural adaptations place chronic stress on the TMJ and masticatory muscles, as with the effects of excessive smartphone use. Additionally, increased cognitive load with prolonged screen exposure can potentially boost psychological tension as well, thus influencing TMD severity by means of central sensitization and neuromuscular impairment. Evidence for laptop-associated musculoskeletal tension has all been in line with neck pain and jaw pain correlations that align closely with the pathophysiology of TMD.

Digital Therapeutics and Future Directions

Emerging EMA tools have introduced real-time monitoring of bruxism, posture, and other functional behaviors, enabling dynamic assessment of temporomandibular activity in naturalistic settings [10]. These smartphone-assisted methods enhance clinical accuracy by continuously tracking patient-specific muscle activity, jaw movements, and posture-related deviations. Furthermore, T-Med platforms and AI-enabled mobile applications have demonstrated promising potential for remote diagnosis, monitoring, and management of TMDs, particularly in post-pandemic clinical workflows [28]. Future research should focus on integrating EMG, kinematic motion tracking, and longitudinal study designs to establish causal pathways between smartphone use and TMD progression. Such integration of advanced digital technologies could pave the way for personalized and preventive therapeutic strategies in TMD care.

Clinical Implications

The findings of this review highlight the growing importance of incorporating ergonomic and behavioral strategies into routine TMD management. Patients should be encouraged to engage in posture correction exercises and ergonomic education to minimize cervical strain and optimize temporomandibular biomechanics. Addressing psychological stress and parafunctional behaviors, such as bruxism and jaw clenching, is equally essential, as these factors amplify muscular tension and pain perception. Finally, the integration of digital behavioral monitoring tools - including smartphone-based feedback systems - into routine clinical follow-up can facilitate continuous assessment, promote adherence to therapeutic exercises, and support long-term prevention of TMD exacerbations.

Limitations

This review was undermined by the dominance of cross-sectional study designs, which restricted the ability to establish causal associations between smartphone use and TMD. Diagnostic instrument heterogeneity for TMD assessment and measurement inconsistency of smartphone use across studies might have impacted results comparability. Lack of electromyographic measurements deprived direct measurement of neuromuscular strain and changes in muscle activity due to smartphone use. Psychosocial and behavioral measures were also measured with self-report questionnaires, which represented a potential risk of reporting bias. Future studies need to incorporate objective measures of posture and muscle activity, standardized measures of smartphone exposure, and longitudinal designs to strengthen the evidence base on smartphone-related risk factors for TMD.

## Conclusions

The overall results provided a noticeable correlation between excessive smartphone use and the severity of TMD, mediated by biomechanical stress, postural deviations, psychological distress, and parafunctional behaviors. Cephalometric analyses and postural assessments in studies proved that forward head posture and cervical misalignment were responsible for greater TMJ loading and masticatory muscle strain. Self-reported stress and smartphone addiction scales also proved a statistically significant correlation between digital dependency and pain-related TMD symptoms. There is moderate evidence that excessive smartphone use contributes to the severity of temporomandibular disorders through biomechanical, postural, and psychosocial mechanisms. Forward head posture and cervical strain amplify TMJ loading, while stress and smartphone addiction intensify parafunctional behaviors. Future longitudinal and interventional studies integrating objective EMG and ergonomic interventions are needed. Preventive strategies should emphasize ergonomic awareness, posture training, and stress reduction in the digital era.

## References

[1] Tran C, Ghahreman K, Huppa C, Gallagher JE (2022). Management of temporomandibular disorders: a rapid review of systematic reviews and guidelines. Int J Oral Maxillofac Surg.

[2] Thomas DC, Singer SR, Markman S (2023). Temporomandibular disorders and dental occlusion: what do we know so far?. Dent Clin North Am.

[3] Busse JW, Casassus R, Carrasco-Labra A (2023). Management of chronic pain associated with temporomandibular disorders: a clinical practice guideline. BMJ.

[4] Kalladka M, Young A, Thomas D, Heir GM, Quek SY, Khan J (2022). The relation of temporomandibular disorders and dental occlusion: a narrative review. Quintessence Int.

[5] David D, Giannini C, Chiarelli F, Mohn A (2021). Text neck syndrome in children and adolescents. Int J Environ Res Public Health.

[6] Maayah MF, Nawasreh ZH, Gaowgzeh RA, Neamatallah Z, Alfawaz SS, Alabasi UM (2023). Neck pain associated with smartphone usage among university students. PLoS One.

[7] Beukenhorst AL, Druce KL, De Cock D (2022). Smartphones for musculoskeletal research - hype or hope? Lessons from a decennium of mHealth studies. BMC Musculoskelet Disord.

[8] Baabdullah A, Bokhary D, Kabli Y, Saggaf O, Daiwali M, Hamdi A (2020). The association between smartphone addiction and thumb/wrist pain: a cross-sectional study. Medicine (Baltimore).

[9] Hwangbo NK, Woo KC, Kim ST (2023). Evaluation of clinical symptoms improvement by cognitive behavioral therapy using a smartphone application in patients with temporomandibular disorder. Healthcare (Basel).

[10] Colonna A, Bracci A, Ahlberg J (2023). Ecological momentary assessment of awake bruxism behaviors: a scoping review of findings from smartphone-based studies in healthy young adults. J Clin Med.

[11] Eitivipart AC, Viriyarojanakul S, Redhead L (2018). Musculoskeletal disorder and pain associated with smartphone use: a systematic review of biomechanical evidence. Hong Kong Physiother J.

[12] Page MJ, Moher D, Bossuyt PM (2021). PRISMA 2020 explanation and elaboration: updated guidance and exemplars for reporting systematic reviews. BMJ.

[13] Igelström E, Campbell M, Craig P, Katikireddi SV (2021). Cochrane's risk of bias tool for non-randomized studies (ROBINS-I) is frequently misapplied: a methodological systematic review. J Clin Epidemiol.

[14] Downes MJ, Brennan ML, Williams HC, Dean RS (2016). Development of a critical appraisal tool to assess the quality of cross-sectional studies (AXIS). BMJ Open.

[15] Divya Lalitha N, Prabu D, Manipal S, Rajmohan M, Bharathwaj VV (2024). Smartphone usage and its association with stress-related bruxism, temporomandibular joint disorder among dental tutees - an analytical investigation. Przegl Epidemiol.

[16] Emodi-Perlman A, Hochhauser T, Winocur P, Friedman-Rubin P, Eli I (2021). The effect of smartphones on daytime sleepiness, temporomandibular disorders, and bruxism among young adults. Quintessence Int.

[17] How QY, Salleh NM, Lim GS (2023). The association of mobile phone addiction and temporo-mandibular disorders (TMD) amongst dental undergraduates, University of Malaya, Malaysia: a pilot study. Malaysian Dental Journal.

[18] Kee IK, Byun JS, Jung JK, Choi JK (2016). The presence of altered craniocervical posture and mobility in smartphone-addicted teenagers with temporomandibular disorders. J Phys Ther Sci.

[19] Mani MS, Ahamed SY, Kavithagiri NL, Ambiga P, Sivaraman G, Balan N (2019). Association of mobile phone usage in patients with temporomandibular joint disorders - a comparative study. Indian J Dent Adv.

[20] Omezli MM, Torul D, Varer Akpinar C (2023). Temporomandibular disorder severity and its association with psychosocial and sociodemographic factors in Turkish adults. BMC Oral Health.

[21] Pei YP, Li HC, Zhong JW, Gao XL, Xiao CQ, Yue Y, Xiong X (2022). The association between problematic smartphone use and the severity of temporomandibular disorders: a cross-sectional study. Front Public Health.

[22] Lim A, Iyer S, Merner B, McCullough M (2023). Smartphone apps for people living with temporomandibular disorder: a systematic review of characteristics, quality, self-management support and user centred design [PREPRINT]. SSRN.

[23] Abdul NS, Kumari M, Shenoy M, Shivakumar GC, Herford AS, Cicciù M, Minervini G (2023). Telemedicine in the diagnosis and management of temporomandibular disorders: a systematic review conducted according to PRISMA guidelines and the Cochrane Handbook for Systematic Reviews of Interventions. J Oral Rehabil.

[24] Borhany T, Shahid E, Siddique WA, Ali H (2018). Musculoskeletal problems in frequent computer and internet users. J Family Med Prim Care.

[25] Susilowati IH, Kurniawidjaja LM, Nugraha S, Nasri SM, Pujiriani I, Hasiholan BP (2022). The prevalence of bad posture and musculoskeletal symptoms originating from the use of gadgets as an impact of the work from home program of the university community. Heliyon.

[26] Young JL, Snell MG, Robles O (2022). Effects of electronic usage on the musculoskeletal system in adolescents and young adults: a systematic review. J Musculoskelet Disord Treat.

[27] Lucka E, Wareńczak-Pawlicka A, Lucki M, Lisiński P (2024). The impact of increased computer screen time during the COVID-19 pandemic on the occurrence of upper part of musculoskeletal diseases among health personnel. Sci Rep.

[28] Synolaki E, Chandolias K, Hristara-Papadopoulou A, Kallistratos I, Mathioudaki A, Antonaki M (2023). The effect of technology on the occurrence of musculoskeletal disorders in students of high school in Greece. Int J Clin Trials.

